# Advances in Detecting Viable/Dead Foodborne Microorganisms Using Diverse Functional Nucleic Acid-Based Molecular Recognition

**DOI:** 10.3390/bios16070364

**Published:** 2026-07-03

**Authors:** Yanger Liu, Huifu Yuan, Juan Zhang, Xiaoyun Sun, Peili Wang, Pazilaiti Yiming, Ailiang Chen, Yanyang Xu

**Affiliations:** 1 State Key Laboratory for Quality and Safety of Agro-Products, Institute of Quality Standards and Testing Technology for Agro-Products, Chinese Academy of Agricultural Sciences, Beijing 100081, China; liuyanger@caas.cn (Y.L.); 15110679191@yeah.net (J.Z.); sxiaoyun2022@163.com (X.S.); peily008@163.com (P.W.); 2 College of Agriculture and Forestry, Hebei North University, Zhangjiakou 075000, China; nkxyhf@163.com; 3 Xinjiang Institute of Veterinary Medicine and Feed Supervision, Urumqi 830063, China; 13609977739@163.com

**Keywords:** FNA, viable bacteria detection, dead bacteria detection, food safety, foodborne pathogens

## Abstract

Accurately detecting viable foodborne pathogenic bacteria is essential for food safety risk assessments and public health interventions. Traditional plate counting is time-consuming and operationally cumbersome. Immunological assays are unable to distinguish viable from dead cells, whereas conventional nucleic acid amplification is often affected by residual DNA originating from dead bacteria. These limitations render conventional approaches inadequate for rapid and precise field detection. Functional nucleic acids (FNAs) offer a promising alternative for viability detection because of their high sensitivity, specificity, target diversity, and programmable integrability. This review provides a systematic overview of molecular recognition strategies and FNA-based detection technologies for identifying viable foodborne microorganisms. We categorize the biomarkers targeted by FNAs into nucleic acids, surface structures, and metabolic activities. Building on this categorization, we examine the core principles and technological evolution of primers, aptamers, DNAzymes, guide nucleic acids, and oligonucleotide probes in viability discrimination. We then outline the practical applications of these technologies across the food supply chain and discuss the remaining challenges and future directions in the field. Ultimately, this work provides a theoretical reference and practical guidance for ensuring food safety and advancing precise microbial risk management.

## 1. Introduction

Foodborne pathogen contamination represents a significant threat to global public health. In 2022, the European Food Safety Authority reported 5763 foodborne disease outbreaks, resulting in 48,605 illnesses, 2783 hospitalizations, and 64 fatalities. This mortality rate is the highest recorded in the past decade [1]. Only viable bacteria with intact cell membranes, active metabolism, and sustained RNA transcription can proliferate in food matrices and cause infections. Dead bacteria, conversely, lack direct pathogenicity due to compromised membrane integrity and metabolic arrest [2,3]. Therefore, accurate discrimination between viable and dead bacteria in a sample is essential for ensuring the validity and reliability of food safety risk assessment and public health interventions [4]. Accordingly, the development of rapid and effective detection strategies for viable bacteria is essential for ensuring food safety [5].

Current bacterial detection methodologies primarily rely on plate counting, immunological assays, and nucleic acid amplification [6]. Plate counting remains the gold standard because it directly distinguishes viable from dead bacteria, as only viable cells can form colonies on selective media [5]. However, its application in rapid field detection is limited by cumbersome procedures and a cultivation period of three to four days [5,6]. Immunological methods like ELISA are rapid and operationally simple, but they rely on antibodies that recognize surface antigens present on both viable and dead cells [6]. Antibodies also suffer from batch-to-batch variation and poor thermal stability [7]. Nucleic acid amplification techniques offer high sensitivity and rapid processing. However, conventional amplification methods cannot eliminate interference from residual DNA of dead bacteria, often resulting in overestimation of viable cell counts [8]. Therefore, there is a clear need for novel molecular recognition technologies that simultaneously achieve high sensitivity and high specificity (Table 1).

Functional nucleic acids (FNAs) have emerged as promising molecular recognition elements to address these limitations [9,10]. FNAs include aptamers, DNAzymes, primer nucleic acids, and guide nucleic acids. They achieve target recognition and signal generation through complementary base pairing or spatial folding [9,10,11]. For viability discrimination, FNAs integrate molecular recognition, signal amplification, and catalytic output, thereby shifting the detection paradigm from passive recognition to active differentiation [12]. The advantages of FNAs stem from several key advantages. Their high sensitivity and specificity enable target recognition at the single-nucleotide or single-cell level. Furthermore, their broad target diversity allows them to bind DNA, RNA, whole cells, metabolic enzymes, and ATP. Owing to their inherent programmability, recognition elements can be rationally designed to target unique molecular features of viable bacteria and can be flexibly coupled with signal amplification modules to suppress background interference from DNA of dead cells. Collectively, these properties make FNAs an ideal platform for developing rapid and precise viability assays.

**Figure 1:**
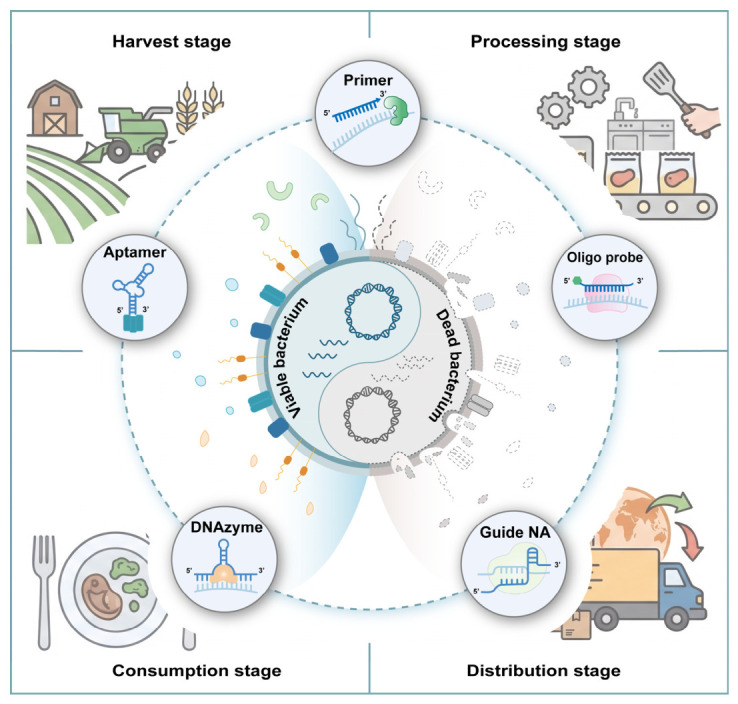
Schematic overview of diverse FNA-based molecular recognition strategies for discriminating viable from dead foodborne bacteria.

Despite recent progress in applying FNAs to bacterial viability detection, comprehensive reviews summarizing these developments are lacking [9,10,11,12]. This review provides a systematic overview of molecular recognition strategies and FNA-based detection technologies for identifying viable foodborne microorganisms. We first outline the mechanisms of viability discrimination based on three core biomarker categories targeted by FNAs: nucleic acids, surface structures, and metabolic activities. We then review the principles and strategies for differentiating viable and dead cells using primer nucleic acids, aptamers, DNAzymes, guide nucleic acids, and oligonucleotide probes. We also summarize the field applications and integration of these technologies across four stages of the food supply chain: raw material production, processing, product circulation, and consumption. Finally, this work examines technical bottlenecks, proposed countermeasures, and future trajectories in the field. Our goal is to provide practical guidance for ensuring food safety and precisely managing microbial risks (Figure 1).

## 2. Biomarkers for Viable/Dead Bacteria Detection Using Diverse FNAs

**Figure 2:**
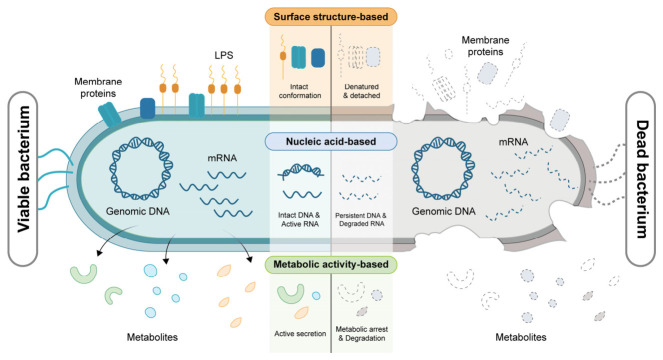
Schematic illustration of the three biomarker categories (surface structure-based, nucleic acid-based, and metabolic activity-related) used for viability discrimination. Viable cells maintain intact spatial conformations, active RNA transcription, and continuous metabolite secretion. Conversely, dead cells exhibit antigen denaturation, rapid RNA degradation, and metabolic arrest. These physiological differences provide specific recognition targets for FNAs.

In viability discrimination, the targets recognized by FNAs span multiple biomarker levels, including nucleic acids, surface structures, and metabolic products. Viable bacteria possess intact cell membranes, active metabolic processes, and continuously transcribed nucleic acids. In contrast, dead bacteria lose these characteristics following membrane damage, metabolic arrest, and nucleic acid degradation. Based on these physiological differences, FNAs achieve live/dead discrimination by targeting specific biomarkers. These biomarkers fall into three main groups based on their physicochemical properties and origins: nucleic acid-based, surface structure-based, and metabolic activity-related markers (Figure 2).

Nucleic acid-based biomarkers represent the most widely recognized targets for FNAs, including genomic DNA, ribosomal RNA (rRNA), and messenger RNA (mRNA). The three nucleic acid targets differ notably in detection performance. Genomic DNA is highly stable with high copy number, supporting high detection sensitivity but no inherent viability discrimination. 16S rRNA has thousands of copies per cell with moderate stability, balancing sensitivity and viability specificity. mRNA has an extremely short half-life and the strongest viability specificity, but its low abundance leads to relatively lower detection sensitivity. Among these, genomic DNA can remain stable in dead bacteria for extended periods. Therefore, DNA detection alone is insufficient to differentiate live from dead bacteria and must be supplemented with membrane integrity dyes or the targeting of RNA possessing a very short half-life. Primer nucleic acids typically target conserved genomic regions in bacteria and facilitate nucleic acid detection through PCR or isothermal amplification [13,14,15,16]. In contrast, guide nucleic acids serve as programmable targeting elements paired with distinct effector systems: gRNA directs CRISPR/Cas systems to cleave target DNA, while gDNA guides Argonaute systems to cleave target RNA [17,18]. Oligonucleotide probes directly target 16S rRNA and achieve specific labeling of viable bacteria through the rapid degradation of rRNA in dead bacteria. Furthermore, mRNA is highly abundant in metabolically active viable bacteria and degrades rapidly within minutes after cell death under most conventional detection conditions, thus providing strong inherent viability discrimination. Hence, reverse transcription primers or gRNA that specifically recognize mRNA sequences can intrinsically distinguish live from dead bacteria [19,20]. Selecting these nucleic acid biomarkers demands careful assessment of their stability, copy number, and species specificity.

Surface structure biomarkers allow the direct capture and recognition of viable bacteria. Common targets include lipopolysaccharides, outer membrane proteins, flagella, and capsular polysaccharides. These molecules maintain their native spatial conformations on viable bacteria but denature or degrade on dead cells. Aptamers isolated via whole-cell SELEX bind these native structures with high affinity, enabling specific cellular enrichment [21]. For example, aptamers targeting viable strains such as *Salmonella* Typhimurium, *Escherichia coli* O157:H7, and *Staphylococcus aureus* have been successfully integrated into various sensing platforms [22,23]. A key advantage of these biomarkers is that they bypass the need for cell lysis, which simplifies and accelerates the detection process.

Metabolic biomarkers serve as the primary targets for DNAzyme-based FNAs. These indicators include extracellular enzymes and metabolites specifically secreted by viable bacteria. Unlike dead cells, metabolically active bacteria continuously produce catalytically active enzymes and small-molecule metabolites. For instance, *Salmonella* secretes ribonuclease RNase H2, which cleaves a specific ribo-adenosine site on a DNAzyme substrate. Similarly, intracellular extracts of *Escherichia coli* contain factors that activate specific RNA-cleaving deoxyribozymes [24]. By targeting these metabolic products, DNAzymes can discriminate viability without relying on membrane integrity or nucleic acid amplification [25]. These biomarkers reflect the actual metabolic state of the bacteria, providing a distinct detection dimension compared to traditional nucleic acid or surface antigen assays. Notably, viable but non-culturable (VBNC) bacteria represent a critical hidden safety risk in the food industry. VBNC cells maintain intact cell membranes and basal metabolic activity, yet lose the ability to form colonies on standard culture media. Consequently, conventional plate counting completely fails to detect this population, and membrane integrity-dependent dyes such as PMA cannot distinguish VBNC cells from culturable viable bacteria, resulting in discrepancies between molecular detection and culture-based gold-standard results. Among FNA-based detection strategies, aptamers recognizing native surface conformations bind effectively to VBNC cells. RNA-targeting amplification and guide nucleic acid systems enable accurate VBNC quantification by detecting persistent rRNA and low-abundance mRNA transcripts. DNAzymes responsive to basal metabolic secretion can also identify VBNC populations with suitable sensitivity. These FNA technologies fill the VBNC detection gap that cannot be addressed by traditional culture methods.

## 3. Primer Nucleic Acid-Based Live/Dead Bacteria Detection Techniques

Primer nucleic acids serve as the core recognition and initiation elements in amplification techniques. They enable exponential nucleic acid amplification by specifically targeting conserved genomic regions under enzymatic action. Currently, primer-based methods for differentiating viable from dead bacteria are primarily divided into thermal cycling amplification and isothermal amplification. This section reviews the principles, strategies, and recent advances of these two approaches.

### 3.1. Primer-Mediated Thermal Cycling Amplification Techniques

Primer nucleic acids are the core components of thermal cycling amplification techniques, predominantly the polymerase chain reaction (PCR). They are typically designed to target conserved regions in bacterial genomes (such as the 16S rRNA and *rpoB* genes) or specific virulence genes, ensuring species-level specificity. DNA polymerase drives exponential target amplification during thermal cycling. These techniques offer high sensitivity, excellent specificity, and reliable quantification, making them standard methods for detecting foodborne pathogens. However, conventional PCR and qPCR cannot discriminate between nucleic acids from viable and dead bacteria. Following cell membrane disruption, the genomic DNA of dead bacteria persists and remains amplifiable, leading to overestimations of viable counts or false-positive signals. Thus, distinguishing viability using thermal cycling amplification requires additional pretreatments or modifications to the target molecules.

Currently, the most widely used strategy combines thermal cycling amplification with photosensitive DNA-binding dyes. Propidium monoazide (PMA) selectively penetrates membrane-compromised dead bacteria and covalently binds DNA, thereby inhibiting PCR amplification. Its optimized derivative PMAxx shows higher suppression efficiency for dead-cell DNA and lower phototoxicity to viable cells, but retains the same membrane-dependent mechanism. Consequently, both PMA and PMAxx perform well for Gram-negative bacteria with thin cell walls but show limited penetration in Gram-positive bacteria and cannot distinguish VBNC cells with intact membranes. In contrast, thiazole orange monoazide (TOMA) is a metabolism-dependent dye that enters all cells and is activated by intracellular esterases in viable bacteria, enabling membrane-independent viability discrimination. It is applicable to both Gram-positive and Gram-negative bacteria and can detect VBNC populations, although it requires longer incubation and is more sensitive to matrix esterase interference. Shi et al. [14] developed a PMA–qPCR assay targeting the *pheS* gene of lactic acid bacteria in fermented milk, enabling absolute quantification of viable cells within 3 h. Results correlated well with plate counting and confocal microscopy and were significantly lower than conventional qPCR, confirming effective removal of dead-cell DNA signals. To improve performance, Huang et al. [26] introduced sodium deoxycholate to enhance PMA penetration into dead cells and incorporated gold nanoparticles (AuNPs) to amplify qPCR fluorescence. The resulting AuNP-SD-PMA-qPCR method detected viable *Listeria monocytogenes* and *Salmonella* at 5 × 10^1^ CFU/g in dairy products after 6 h enrichment. Liu et al. [27] further integrated PMA into multiplex PCR for simultaneous detection of viable *Escherichia coli* O157:H7, *Staphylococcus aureus*, and *Salmonella*, achieving a detection limit of 10^4^ CFU/mL and complete suppression of dead-cell signals when the viable-to-dead ratio exceeded 1:10.

**Figure 3:**
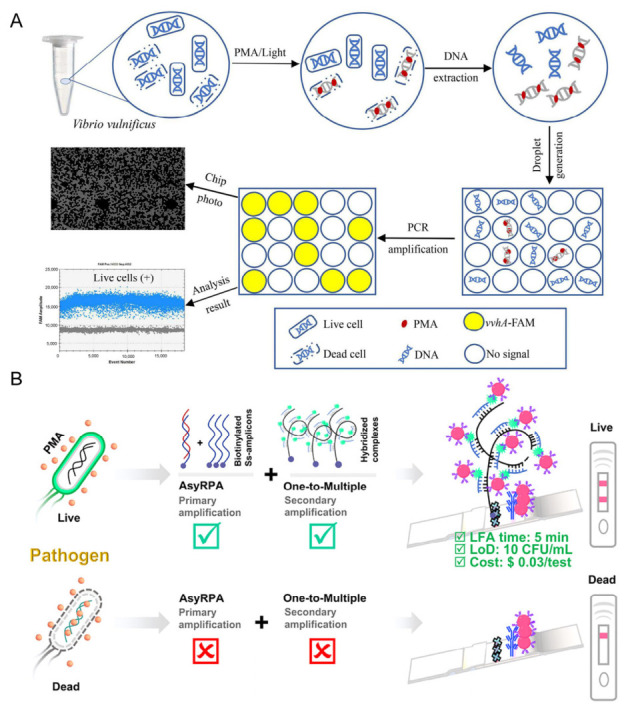
Schematic overview of primer-based amplification strategies for viability discrimination. (**A**) A PMA-ddPCR strategy integrating photosensitive dye pretreatment with droplet digital PCR for the highly sensitive and absolute quantification of viable *Vibrio vulnificus* [28], with permission from Frontiers, 2022. (**B**) An isothermal amplification method combining photosensitive dye treatment with RPA and lateral flow strips for rapid visual detection [31], with permission from the American Chemical Society, 2026.

Although PMA-qPCR is widely used, its quantification depends on standard curves, and its sensitivity for low-abundance targets is limited by amplification efficiency and background interference. Droplet digital PCR (ddPCR) partitions the reaction system into tens of thousands of independent droplets to achieve absolute quantification. It eliminates the reliance on standard curves and exhibits better tolerance to PCR inhibitors. Hu et al. [28] developed a PMA-ddPCR method targeting the *vvhA* gene of *Vibrio vulnificus*, attaining a detection limit of 29.33 CFU/mL in pure culture and 65.20 CFU/mL for simulated plasma samples, representing a 15- to 40-fold improvement in sensitivity relative to PMA-qPCR (Figure 3A). Yang et al. [15] developed a triplex PMA-ddPCR approach to simultaneously identify *Vibrio cholerae* O1 and O139 serogroups and the cholera enterotoxin *ctxA* gene. They achieved detection limits in seawater of 150.66 CFU/mL for O1, 147.57 CFU/mL for O139, and 2 copies per reaction for *ctxA*. This approach effectively discriminated mixed populations of live and heat-killed bacteria, yielding results comparable to plate counting. These studies demonstrate that PMA-ddPCR offers distinct advantages over qPCR, particularly for detecting low-abundance viable bacteria in complex matrices. Notably, PMAxx, an improved variant of PMA, is also fully compatible with conventional PCR, qPCR and ddPCR platforms [15,29,30]. With reduced phototoxicity and more effective suppression of residual DNA from dead cells, PMAxx has been increasingly adopted in thermal cycling amplification assays for bacterial viability discrimination.

A dye-free strategy takes advantage of the extremely short half-life of mRNA (minutes) in viable bacteria compared to the long-lasting stability of DNA, allowing for the direct detection of viability-specific transcripts via reverse transcription PCR (RT-PCR). This approach inherently discriminates viability and eliminates the need for chemical dye treatments. Zhou et al. [20] coupled splintR ligase with PCR and CRISPR/Cas12a to target the RNA of the *Salmonella hilA* gene. A ligation reaction converted the RNA signal into a DNA signal, followed by PCR amplification and Cas12a trans-cleavage to yield a three-level cascade signal amplification. This method achieved a detection limit of 10 CFU and effectively discriminated viable from dead bacteria. However, RNA is prone to degradation, and the required reverse transcription step imposes operational demands.

### 3.2. Primer-Mediated Isothermal Amplification Techniques

Primer nucleic acids are also central to isothermal amplification techniques. Because of different amplification mechanisms, primer design for isothermal methods differs from thermal cycling, where primers typically target a single short sequence. For example, loop-mediated isothermal amplification (LAMP) usually requires 4 to 6 primers that recognize 6 to 8 distinct regions within the target gene. In contrast, recombinase polymerase amplification (RPA) and recombinase-aided amplification (RAA) rely on recombinase-mediated primer-template binding. This approach simplifies primer design to just 2 primers but demands greater stringency regarding length and melting temperature (Tm) values. Isothermal amplification uses strand-displacing DNA polymerases to achieve exponential amplification at a constant temperature, eliminating the need for precise thermal cycling instruments and making it highly suitable for rapid on-site detection. However, isothermal amplification alone cannot directly determine viability and thus requires auxiliary strategies.

Photosensitive dye-based strategies are widely used, with PMA commonly combined with isothermal amplification methods such as LAMP, RPA, RAA, and strand exchange amplification (SEA) [31] (Figure 3B). The improved derivative PMAxx has largely replaced PMA due to stronger suppression of dead-cell DNA amplification, reduced phototoxicity, and minimal effects on viable cell membranes. Wen et al. [13] developed a PMAxx-LAMP assay targeting the *rfbE* gene of *Escherichia coli* O157:H7. Treatment with 6 μM PMAxx for 5 min fully inhibited amplification from 10^6^ CFU/mL dead cells. Coupled with immunomagnetic separation (IMS), the method achieved a detection limit of 81 CFU/g in lettuce within 2 h. Li et al. [16] further integrated IMS, PMAxx-LAMP, and a lateral flow strip into a portable platform for on-site *Salmonella* detection, reaching 10^2^ CFU/mL in milk without interference from dead bacteria. However, PMA-based approaches rely on membrane integrity, limiting performance for Gram-positive bacteria, complex membrane structures, and VBNC cells. To overcome this, Feng et al. [32] developed TOMA, a photosensitive dye that targets metabolic activity. TOMA penetrates all cells but is hydrolyzed by intracellular esterases in viable bacteria, preventing DNA crosslinking; in dead cells, it binds DNA and blocks amplification. Coupled with RAA, TOMA enabled *Salmonella* detection with limits of 2 × 10^4^ CFU/mL in pure culture and 3.5 CFU/mL in milk after enrichment, and was further applied to *Klebsiella pneumoniae* detection in infant formula (2.3 × 10^4^ CFU/g) [33]. By relying on metabolic activity rather than membrane integrity, TOMA offers improved theoretical accuracy and better suitability for complex matrices.

Dye-free isothermal strategies for viable bacteria detection have also advanced. Because mRNA exists exclusively in viable cells, methods like RT-LAMP and RT-RPA provide inherent viability discrimination. Xue et al. [19] coupled nucleic acid sequence-based amplification (NASBA) with the CRISPR/Cas13a system to target *Salmonella* 16S rRNA. Their cNASBA method achieved a detection limit of 1.5 CFU and accurately quantified viable bacteria making up as little as 1% of a mixed population. Furthermore, exploiting the specific infection of viable bacteria by bacteriophages, Xiao et al. [34] integrated phage amplification with SEA and the CRISPR/Cas12a system to build a detection platform that bypasses nucleic acid extraction. This platform provided a sensitivity of 10^3^ CFU/mL for *Pseudomonas aeruginosa* in urine, matching clinical results. Qi et al. [35] developed a finger-actuated microfluidic chip integrating IMS, PMAxx treatment, magnetic DNA extraction, and RAA detection. The chip achieves a detection limit of 130 CFU/mL for *Salmonella* in chicken samples. It supports real-time fluorescence signal analysis via a smartphone app, realizing truly rapid on-site detection (Table 2).

## 4. Aptamer-Based Live/Dead Bacteria Detection Techniques

Aptamers are single-stranded oligonucleotides selected through systematic evolution of ligands by exponential enrichment (SELEX). They fold into specific three-dimensional conformations, enabling high-affinity and high-specificity binding to target molecules. Relative to antibodies, aptamers offer high chemical stability, low synthesis costs, minimal batch-to-batch variation, ease of modification, and the capacity for in vitro selection. The unique value of aptamers for viable bacteria detection stems from their requirement for native target conformations [41,42,43]. On viable bacteria, surface structures including lipopolysaccharides, outer membrane proteins, and flagella remain intact with correct spatial folding. Upon death, these antigens denature or are shed, preventing effective aptamer binding. This property enables the inherent discrimination of viable from dead bacteria, eliminating the need for chemical dyes targeting membrane integrity or nucleic acid amplification steps.

Whole-cell SELEX is the primary strategy for generating aptamers against viable bacteria [42]. It employs intact live cells as targets and enriches high-affinity sequences through iterative cycles of binding, separation, and amplification. A key step is counter-selection using non-target strains and dead bacteria, which removes sequences binding to denatured surface epitopes and ensures specificity toward native surface conformations of viable cells. Using this strategy, aptamers have been developed for multiple foodborne pathogens, including *Salmonella* Typhimurium [23], *Escherichia coli* O157:H7 [22], *Staphylococcus aureus* [21], and *Pseudomonas aeruginosa* [43]. For example, Liu et al. [44] identified aptamers V8 and V13 targeting viable and VBNC *Vibrio vulnificus*, with Kd values of 11.22 nM and 15.22 nM, respectively, and a detection limit of 29.96 CFU/mL by flow cytometry. Notably, this was the first report enabling simultaneous detection of viable and VBNC bacteria, addressing a key limitation in conventional approaches. Recent advances in SELEX design further improve performance. Du et al. [45] incorporated food matrices (pork, chicken, milk, beef) as counter-selection environments, yielding anti-matrix-interference aptamers for *S.* Typhimurium. The ROU-24 aptamer achieved 94.2–110.7% recovery in real samples, outperforming buffer-based selections. Pham et al. [43] used an Eppendorf tube-based SELEX system with viable bacteria as the target and isolated the highly selective *Pseudomonas aeruginosa* aptamer T1 after 10 rounds of selection. Currently, emerging technologies, including flow cytometry-assisted SELEX [46], three-dimensional structure-assisted SELEX [47], and microfluidic chip system-assisted SELEX [48], are steadily enhancing the efficiency and specificity of whole-cell SELEX [42].

Integrating aptamers with CRISPR-Cas systems has recently attracted considerable attention. In these platforms, aptamers specifically capture viable bacteria and release activator strands via conformational changes or strand displacement reactions, which subsequently trigger the trans-cleavage activity of CRISPR-Cas. These technologies have evolved from amplification-dependent to amplification-free detection and from qualitative to highly sensitive quantitative analysis. Early approaches primarily relied on releasing activator strands through strand displacement to activate CRISPR-Cas trans-cleavage. Geng et al. [49] reported an aptamer-CRISPR/Cas12a detection platform directed at viable *Shigella flexneri*. In this design, a blocker strand complementary to the aptamer was displaced upon specific aptamer binding to viable bacteria. The liberated blocker then activated Cas12a to cleave a fluorescent reporter probe, yielding a detection limit of 225 CFU/mL within 1.5 h and effectively discriminating between viable and heat-inactivated bacteria. Later, Zhang et al. [22] constructed a spherical nucleic acid AuNP-aptamer-triggered CRISPR/Cas12a system. This system employed poly-T-templated copper nanoparticles as the fluorescence reporter for an amplification-free PTCas12a platform targeting *Staphylococcus aureus*. It delivered a detection limit as low as 3.0 CFU/mL with a dynamic range spanning 1.0 × 10^1^ to 1.0 × 10^6^ CFU/mL. More recently, Geng et al. [50] combined a dual-aptamer system with CRISPR/Cas14. One aptamer facilitated magnetic bead enrichment, while the other activated Cas14. Using the ability of Cas14 to recognize ssDNA independently of a protospacer adjacent motif (PAM) sequence, this approach attained a detection sensitivity of 10 CFU/mL within 15 min at room temperature without nucleic acid extraction or amplification. When assessed on clinical samples of periprosthetic joint infection, the method yielded a positive agreement rate of 83.3% and a negative agreement rate of 100%. Together, these investigations illustrate the rapid progress of aptamer-CRISPR technology, advancing the achievable detection sensitivity from the micromolar range down to the femtomolar range.

**Figure 4:**
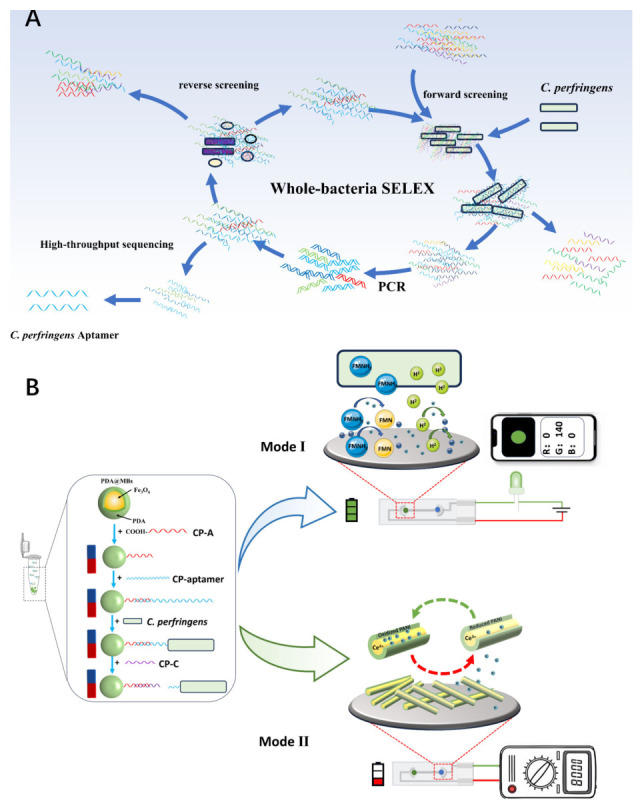
Schematic diagram of a bipolar electrode (BPE) aptasensor designed for the rapid detection of common foodborne pathogens based on cellular respiratory chain activity [41], with permission from the American Chemical Society, 2026. (**A**) Screening workflow of *Clostridium perfringens*-specific aptamer via whole-bacteria SELEX, consisting of reverse screening, forward screening and high-throughput sequencing. (**B**) Schematic illustration of the dual-mode sensing mechanism: Mode I shows the construction of PDA@MBs-aptamer capture probes; Mode II demonstrates the target binding and bipolar electrode signal output process.

Beyond CRISPR systems, aptamers can be integrated into liquid crystal, SERS, electrochemical, and microfluidic platforms to enable rapid, sensitive, and portable detection across diverse applications. Lateral flow chromatography achieves on-site detection by replacing antibodies with AuNP–aptamer conjugates, enabling visual detection at 2.34 × 10^2^ CFU/mL within 15 min without complex instrumentation [43]. To enhance sensitivity and functionality, Xie et al. [51] developed a SERS/ATP-bioluminescence dual-mode immunosensor based on a sandwich structure (capture probe–bacteria–signal probe). SERS quantifies total bacteria, while LED-induced photothermal lysis releases ATP for bioluminescence-based viable cell detection; dead bacteria are calculated by subtraction, enabling triple-mode analysis in one tube. Yang et al. [41] utilized extracellular electron transfer of viable bacteria to construct a reusable bipolar electrode (BPE)-based dual-mode aptasensor (Figure 4). The system supports 12 reuses, achieves a 2.50 CFU/mL detection limit, 3 min assay time, and <$1 cost per test, with matrix effects <7% in chicken and milk samples. Zhang et al. [22] combined aptamer-functionalized magnetic nanoparticle enrichment with droplet microfluidics for single-bacterium encapsulation. IPTG induction triggers β-galactosidase secretion, which hydrolyzes FDG to generate fluorescence, enabling single-cell quantification of viable *Escherichia coli* O157:H7 with a 10 CFU/mL detection limit and 3 h total assay time. Overall, these studies demonstrate that aptamer-based sensing systems are advancing toward rapid, sensitive, portable, and low-cost detection, offering broad applications in food safety, environmental monitoring, and clinical diagnostics (Table 3).

## 5. DNAzyme-Based Live/Dead Bacteria Detection Techniques

DNAzymes are single-stranded DNA molecules that combine recognition capabilities with catalytic activity [7,9]. They primarily bind to metabolites or extracellular enzymes secreted exclusively by viable bacteria. Because dead bacteria cease metabolic function and do not produce these molecular targets, DNAzymes can inherently discriminate live from dead cells. This mechanism eliminates the need to assess membrane integrity or amplify nucleic acids, making DNAzymes highly advantageous for viability detection.

Recognizing viability-specific metabolites is the primary mechanism by which DNAzymes distinguish live from dead cells. For example, metabolically active *Salmonella* secretes the ribonuclease RNase H2, which highly specifically cleaves a single ribo-adenosine site within the substrate strand of a DNAzyme. Wu et al. [24] applied this mechanism to develop an epDNAzyme detection platform based on a sequential reporting strategy. The platform decouples substrate cleavage from signal output. After cleavage, a segmented reporter probe is introduced to generate a fluorescence signal through proximity hybridization. This design prevents the inhibition of catalytic activity often caused by internal fluorescent modifications in conventional DNAzymes. It increases the catalytic rate constant by approximately 20-fold, detects viable *Salmonella* down to 190 CFU/mL within 25 min, and identifies a viable bacterium proportion as low as 0.1% in a mixed population. Deng et al. [25] later combined this mechanism with a Z-scheme g-C_3_N_4_/V_2_C heterojunction photoelectric material to construct a DzPEC photoelectrochemical sensor. In this system, DNAzyme cleavage triggers the release of an SiO_2_ photoquencher and restores the photocurrent output of the heterojunction. The sensor achieved a detection limit of 141 CFU/mL, a processing time of 20 min, reliable quantification of viable bacteria at proportions as low as 0.1%, and recovery rates of 95.6% to 98.6% in real samples such as beef, milk, and blood.

**Figure 5:**
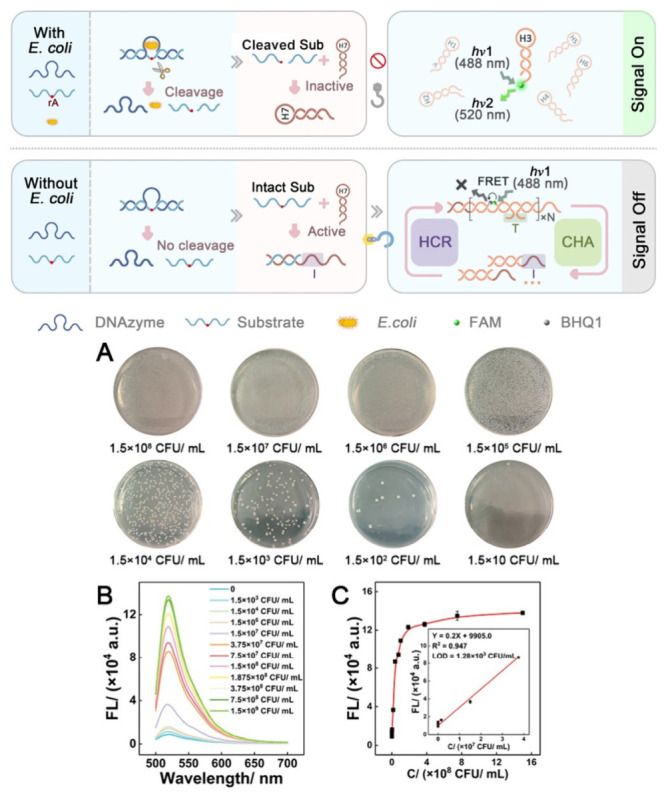
Hook-shaped DNAzyme-based autocatalytic DAHA nucleic acid circuit for fluorescent *Escherichia coli* detection. The top schematic illustrates the sensing mechanism: DNAzyme only cleaves substrate strands upon incubation with viable *E. coli*, releasing trigger fragments to initiate coupled HCR-CHA cascade amplification and generate detectable FAM fluorescence; without target bacteria, intact substrates maintain FRET quenching and produce no fluorescence signal. (**A**) Colony counting plates of serially diluted *E. coli* samples. (**B**) Fluorescence emission spectra of the DAHA biosensor under gradient concentrations of *E. coli*. (**C**) Fluorescence intensity response curve corresponding to increasing bacterial loads, with the inset showing the linear calibration relationship for quantitative detection [54]. Copyright 2024 American Chemical Society.

To improve sensitivity and field applicability, DNAzyme assays have transitioned from standalone platforms to integrated systems. You et al. [53] combined the strain-level specificity of bacteriophages with the catalytic activity of DNAzymes to create a Phage@DNAzyme probe. By immobilizing approximately 56 DNAzyme molecules on each phage surface, they achieved integrated capture and catalysis. This method yielded a detection limit of 50 CFU/mL and was successfully applied to *Salmonella* Typhimurium detection. Furthermore, integrating DNAzymes with enzyme-free nucleic acid amplification circuits can significantly enhance signal output. Liu et al. [54] developed a nucleic acid circuit autocatalytically activated by a hook-shaped DNAzyme (Figure 5). Upon bacterial recognition, the DNAzyme cleaves its substrate, preventing the activation of a “hook” connector hairpin. This halts the subsequent hybridization chain reaction and catalytic hairpin assembly, thereby avoiding fluorescence quenching and producing a sensitive signal on readout. This platform achieved a detection limit of 1.3 × 10^3^ CFU/mL and demonstrated universal applicability for detecting various pathogens simply by replacing the DNAzyme recognition domain. 

Integrating DNAzymes with techniques like LAMP and CRISPR/Cas has also broadened their application scope. Sewid et al. [55] integrated DNAzymes with LAMP by embedding DNAzyme sequences within the amplified products. These amplicons were then detected via a DNAzyme-catalyzed colorimetric reaction, reaching a detection limit below 6.3 CFU. In CRISPR-based strategies, DNA fragments generated by DNAzyme cleavage can serve as activator strands to trigger the trans-cleavage activity of Cas12a. Alternatively, a DNAzyme cascade can be used to transiently inhibit the CRISPR/Cas12a system; an active DNAzyme cleaves a hairpin structure, restoring CRISPR/Cas activity. This approach enables the ultrasensitive detection of *Staphylococcus aureus* without requiring nucleic acid extraction or amplification (Table 4).

## 6. Guide Nucleic Acid-Based Live/Dead Bacteria Detection Techniques

Guide nucleic acids are programmable recognition molecules that direct effector enzymes to specifically cleave target sequences or generate signals through complementary base pairing [9,17]. Depending on the nucleic acid type, they primarily fall into two categories: gRNA, which mediates the CRISPR/Cas system, and gDNA, which mediates the Argonaute system. Both have distinct characteristics for differentiating viable and dead bacteria. CRISPR/Cas systems use RNA-guided effector proteins, require specific PAM sequences for target recognition, typically function at 37 °C, and benefit from mature design tools. Argonaute systems utilize DNA guides and operate without PAM requirements, enabling more flexible target selection. However, most thermophilic variants require elevated reaction temperatures and currently have comparatively limited established design resources. This section details the core principles and recent applications of these two systems in viability discrimination.

### 6.1. gRNA-Mediated Crispr/Cas Systems

The gRNA is the core nucleic acid element for target recognition in CRISPR/Cas systems. It typically consists of a spacer sequence, which is complementary to the target nucleic acid, and a scaffold sequence that binds the Cas protein. By designing the spacer region, one can program CRISPR/Cas systems to recognize specific nucleic acid sequences, offering significant potential for molecular detection. For viability discrimination, CRISPR/Cas strategies generally follow two main approaches. The first approach targets DNA using gRNA combined with PMA pretreatment, thereby distinguishing viable from dead cells. The second approach targets RNA directly. Because RNA remains intact in viable bacteria but degrades rapidly in dead cells, this strategy provides inherent discrimination without chemical pretreatments. Furthermore, multiplexing gRNAs allows for the simultaneous detection of both total and viable bacteria.

RNA-targeting strategies have attracted considerable interest because of their inherent discriminatory capability. Viable bacteria maintain an active metabolism, exhibiting continuous transcription and a high abundance of intracellular RNA species, such as 16S rRNA and mRNA. Conversely, dead bacteria cease metabolic activity, and their RNA is rapidly degraded by endogenous nucleases. Directing Cas13a toward RNA targets via gRNA enables the specific recognition of viable bacteria without chemical dyes. Zhang et al. [18] designed a crRNA complementary to the 16S rRNA of *Bacillus cereus*. They replaced the conventional fluorophore-quencher RNA reporter with the light-up RNA aptamer Broccoli. This substitution yielded a one-pot, mix-and-read platform that required no reverse transcription, nucleic acid amplification, or chemical labeling. It achieved a detection limit of 10 CFU and accurately quantified viable bacteria making up as little as 1% of a 10^5^ CFU total bacterial population. To address complex matrix interference in fermented foods, Xu et al. [56] developed a Cas13a-Csm6 tandem nuclease system. Here, Cas13a recognizes the target RNA and cleaves an F-A_4_U_6_ substrate, producing rA_4_>p. This product activates Csm6, which then cleaves a fluorescent reporter probe, resulting in cascade signal amplification. This tandem approach improved sensitivity 16-fold compared to Cas13a alone and identified 1% viable bacteria within 30 min. The method successfully monitored viable lactic acid bacteria and *Bacillus* dynamically during Baijiu jiuqu fermentation.

**Figure 6:**
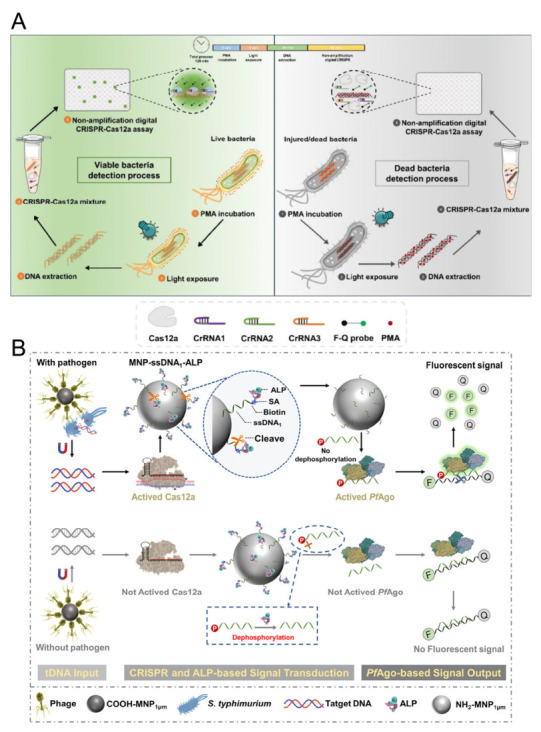
Schematic representation of programmable guide nucleic acid-based strategies for viability discrimination. (**A**) An amplification-free digital CRISPR/Cas12a microfluidic assay coupled with PMA pretreatment for the absolute quantification of viable bacteria [57], with permission from the American Chemical Society, 2024. (**B**) A dual-enzyme cascade signal amplification platform integrating phage-mediated lysis, CRISPR/Cas12a, and PfAgo for highly sensitive viable pathogen detection [59], with permission from the American Chemical Society, 2023.

Targeting DNA alongside PMA pretreatment is another well-established strategy. This approach relies on PMA selectively entering dead bacteria and covalently crosslinking to their DNA. The modified DNA evades Cas protein recognition, ensuring that only DNA from viable bacteria triggers the CRISPR/Cas signal. Yin et al. [57] first paired PMA with CRISPR/Cas12a to establish an amplification-free digital CRISPR microfluidic platform (Figure 6A). They demonstrated that PMA-modified DNA failed to activate Cas12a trans-cleavage and that hydroxylamine compounds from PMA photolysis did not affect Cas activity. By using microwell confinement, the platform reduced the reaction time to 15 min, suppressed 99% of the interference from dead bacteria, and achieved a detection limit of 3.6 × 10^4^ CFU/mL. The team later developed a PMA-assisted, amplification-free digital CRISPR/Cas12a platform that achieved absolute quantification via single-molecule counting, removing the need for standard curves. It provided a detection limit of 1.2 × 10^3^ CFU/mL and a Pearson correlation coefficient of 0.9490 with plate counting across 21 real samples. Additionally, Yin et al. [58] developed a digital CRISPR microfluidic platform integrating PMA with a microfluidic chip. By optimizing the microwell diameter (50 μm) to enhance relative nucleic acid concentrations, they reduced qualitative detection time to 5 min and absolute quantification time to 15 min.

Dual CRISPR systems enable the simultaneous one-tube detection of both total and viable bacteria. Jiao et al. [60] developed a dual CRISPR system targeting DNA and RNA, based on the principle that DNA persists after cell death while RNA degrades rapidly. Cas12a recognizes target DNA (indicating total bacteria), while Cas13a recognizes target RNA (indicating viable bacteria). These events trigger distinct Raman signals, providing a detection limit of approximately 10 CFU/mL in 45 min. This strategy allows multiplexed signal encoding via SERS, avoiding the spectral overlap typical of fluorescence detection. Optimizing gRNA design is crucial for maximizing CRISPR-based detection performance. Xu et al. [56] systematically screened ten crRNAs targeting the 16S rRNA of lactic acid bacteria and ten targeting *Bacillus*. Recognition efficiency varied significantly. Only crRNA-PP-3 and crRNA-BL-1 effectively activated the cascade reaction, with signal-to-background ratios of 2.66 and 3.27, respectively. This highlights that gRNA target site selection requires careful consideration of RNA secondary structure, spacer length, and GC content. Furthermore, Yin et al. [58] improved the sensitivity of amplification-free CRISPR detection more than 10-fold by combining three crRNAs targeting distinct regions of the *rfbE* gene, demonstrating the effectiveness of a multi-gRNA strategy.

### 6.2. gDNA-Mediated Argonaute Systems

The gDNA is the central recognition element of the Argonaute (Ago) system. Typically a 5′-phosphorylated single-stranded DNA of 16 to 21 nucleotides, it directs the Ago protein to specifically cleave target DNA via complementary base pairing. Compared to gRNA in CRISPR systems, gDNA offers higher chemical stability, lower synthesis costs, and independence from a PAM sequence, providing greater flexibility in target selection. Based on the optimal reaction temperature of the Ago protein, these systems are primarily classified into mesophilic and thermophilic Ago. This section outlines the principles and applications of these two gDNA-Ago classes for viability detection.

The “two-step cleavage” mechanism of mesophilic Argonaute (CbAgo) provides a simple and efficient strategy for viable bacteria detection. CbAgo from *Clostridium butyricum* exhibits high cleavage activity at mesophilic temperatures (20 to 37 °C), avoiding the high-temperature requirement (>65 °C) of most pAgos. Zhao et al. [17] applied this property of CbAgo by using bacteriophage LPST10 to specifically capture viable *Salmonella* Typhimurium. After cell lysis with radioimmunoprecipitation assay buffer, DNA was directly released. Three complementary 16 nt gDNAs were designed to guide CbAgo in a primary cleavage of the target DNA, producing 16 nt tDNA molecules. These tDNAs acted as secondary gDNAs, directing a second cleavage step that cut a biotinylated fluorescent probe. The cleaved probes were immobilized on a single polystyrene microsphere via streptavidin, and a machine vision algorithm quantified the signal. This method achieved a detection limit of 40.5 CFU/mL and a linear range of 50 to 10^7^ CFU/mL, with signals originating exclusively from viable bacteria. The workflow requires neither DNA extraction nor amplification, highlighting the convenience and sensitivity of the gDNA-Argonaute system.

Thermophilic Argonaute (PfAgo) enhances detection performance through multi-target synergy and cascade amplification. PfAgo from *Pyrococcus furiosus* operates optimally at 95 °C, and its two-step cleavage capability makes it an ideal tool for generating amplified signals without target amplification. Wang et al. [61] developed a PfAgo platform activated synergistically by DNA and ATP. In this system, bacteriophages lysed viable *Salmonella*, releasing both DNA and ATP. The ATP displaced gDNA_5_ from a bivalent aptamer, allowing it to assemble with PfAgo and cleave a fluorescent probe. Simultaneously, the released DNA recruited PfAgo via three gDNAs to execute the two-step cleavage. Both signaling pathways operated simultaneously in a single tube, markedly improving sensitivity over single-trigger methods. The platform detected as few as 20 CFU/mL without DNA extraction or amplification. Chen et al. [59] integrated CRISPR/Cas12a with PfAgo in a dual-enzyme cascade signal amplification platform (Figure 6B). Phage-mediated lysis released DNA that activated Cas12a. Cas12a then cleaved ssDNA-ALP immobilized on magnetic beads. The remaining ALP dephosphorylated P-gDNA, reducing the substrate pool available for PfAgo activation. This design produced a signal inversely correlated with target concentration. By exploiting the trans-cleavage activity of Cas12a and the specific cleavage of PfAgo, this method achieved a detection limit of 23 CFU/mL and was successfully validated in real food samples (Table 5).

## 7. Oligonucleotide Probe-Based Live/Dead Bacteria Detection

Oligonucleotide probes are functional nucleic acids designed based on complementary base pairing. Unlike amplification-dependent primer nucleic acids, they are direct hybridization-based recognition elements that generate detectable signals via covalently attached labels without enzymatic amplification, belonging to non-amplification FNA detection strategies. They specifically recognize 16S rRNA sequences in target microorganisms and produce visual signals through covalently attached fluorophores, such as Alexa Fluor 488 and Alexa Fluor 647. Unlike techniques relying on signal amplification, these probes directly bind to highly abundant ribosomal RNA within intact cells. This direct recognition enables specific labeling without requiring nucleic acid extraction or amplification. For viability discrimination, this technique commonly incorporates PMA. PMA selectively penetrates dead bacteria to label their DNA, while the fluorescent probe marks the 16S rRNA of all target cells. Dual-channel flow cytometry (FCM) then simultaneously acquires total and dead bacterial counts, allowing for the direct calculation of the viable population. This strategy offers high-throughput detection through one-step staining and dual-parameter quantification.

**Figure 7:**
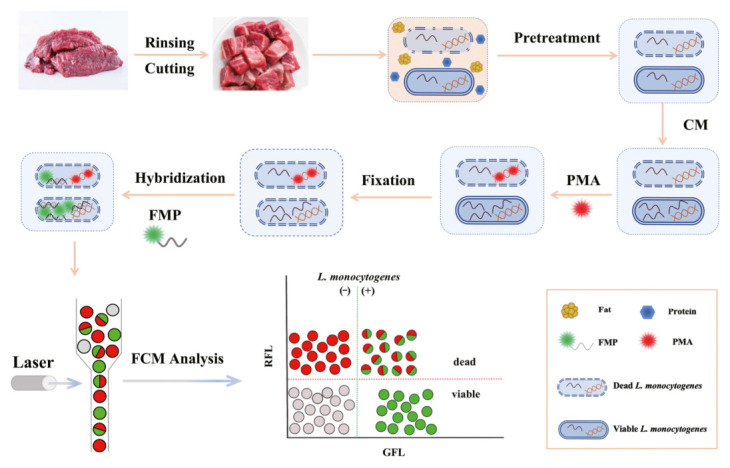
Oligonucleotide probe-based detection techniques for direct viability discrimination [64], with permission from Elsevier, 2025.

Fluorescent probes targeting 16S rRNA are essential for species-level discrimination. Hypervariable regions within 16S rRNA sequences retrieved from databases typically serve as probe targets, with specificity confirmed through Basic Local Alignment Search Tool alignment and experimental validation. Cai et al. [63] developed two probes, L-NOP (labeled with Alexa Fluor 647) and S-NOP (labeled with Alexa Fluor 488), targeting the V1-V2 region of 16S rRNA to detect *Lactobacillus* and *Streptococcus thermophilus* in yogurt. By coupling PMA staining with flow cytometry, they simultaneously quantified viable cells of both probiotics in under 2 h. Testing across 48 strains confirmed the species specificity of both probes, yielding a linear correlation coefficient of 0.99 when compared to plate counting. Liu et al. [64] designed the FMP RL-2 probe to target the 1275–1294 region of *Listeria monocytogenes* 16S rRNA (Figure 7). They combined this probe with PMA treatment to rapidly quantify viable bacteria in beef. To reduce the hybridization time to 30 min, they used chloramphenicol pretreatment to increase the rRNA copy number in viable cells. The detection limit reached 10^2^ CFU/g with an R^2^ value of 0.9994 relative to plate counting, and the method accurately discriminated mixed populations of viable and dead bacteria.

Optimizing probe hybridization, PMA treatment efficiency, and FCM gating strategies is critical for this technique. Wang et al. [65] systematically optimized the hybridization temperature, probe concentration, fixation time, and hybridization time for the Lab 158 probe to detect lactic acid bacteria in infant formula. They introduced chloramphenicol pretreatment to enhance rRNA signals and confirmed that DNA-targeting PMA and RNA-targeting oligonucleotide probes operate without mutual interference. This combination allowed accurate quantification of viable bacteria within 2.5 h, reaching a detection limit of 2.7 × 10^4^ cells/g. For FCM analyses, dual-parameter scatter plots are typically used for logical gating to eliminate background noise from dead bacteria and non-target particles. Bai et al. [66] optimized PMA concentration and exposure time for *Bacillus cereus* detection, integrating these steps with FISH staining using the pB394 probe. This method quantified viable bacteria in meat products and milk powder within 1.5 h, with recovery rates between 96.3% and 97.7%. By evaluating samples with varying live-to-dead ratios, they demonstrated that the PMA-FISH-FCM method accurately captures the viable proportion, matching plate counting results (Table 6).

## 8. Applications

Detection technologies using multiplexed functional nucleic acids for viability discrimination show broad application prospects in foodborne pathogen surveillance. These advancements have facilitated the development of numerous detection devices suitable for field deployment, complex matrices, and resource-limited scenarios across the entire food supply chain. Key application stages include raw material harvest, processing, product distribution, and consumption.

### 8.1. Harvest Stage

Monitoring viable bacteria during raw material harvest faces the dual challenges of complex matrices (such as seawater, river water, and raw milk) and generally low target concentrations. Moreover, it requires eliminating background interference from abundant dead environmental bacteria. Technologies applied at this stage must therefore offer high sensitivity, absolute quantification, and straightforward field operability. For water samples, Zhang et al. [22] integrated aptamer-functionalized magnetic nanoparticles with droplet microfluidics to achieve absolute single-cell quantification of *Escherichia coli* O157:H7. This approach attained a detection limit of 10 CFU/mL and enabled the direct analysis of environmental water without prior enrichment. Similarly, Yang et al. [15] used a PMA-ddPCR assay to simultaneously detect *Vibrio cholerae* serogroups O1 and O139 and the *ctxA* gene in seawater, effectively eliminating dead cell interference. For raw milk monitoring, Li et al. [16] developed a portable toolbox combining immunomagnetic separation with nucleic acid lateral flow assays for rapid on-site *Salmonella* detection. This system achieved a detection limit of 10^2^ CFU/mL without being affected by high concentrations of dead bacteria. Together, these technologies provide powerful tools for controlling pathogen contamination at the source.

### 8.2. Processing Stage

Food processing involves washing, cutting, heat treatment, and the temporary storage of partially processed products, all of which introduce substantial risks of cross-contamination. Because these samples frequently contain PCR inhibitors like fats and proteins, detection technologies must provide strong matrix interference resistance and rapid response times. To target viable bacteria in raw materials like lettuce and chicken, Wen et al. [13] coupled PMA-assisted LAMP with immunomagnetic separation to suppress dead bacterial background signals. They achieved a detection limit of 81 CFU/g for *Escherichia coli* O157:H7 in lettuce within 2 h. Qi et al. [35] designed a manually actuated microfluidic chip that integrates enrichment, processing, extraction, and detection. By using a smartphone application for continuous fluorescence analysis, this device enabled rapid at-line screening. Additionally, Deng et al. [25] combined a *Salmonella*-specific DNAzyme with photoelectric materials to construct a sensor exhibiting recovery rates of 95.6% to 98.6% in beef and milk. Requiring only 20 min, the assay enables rapid evaluation of bactericidal efficacy. These strategies collectively improve the reliability and timeliness of viability detection during food processing.

### 8.3. Distribution Stage

Product distribution involves batch sampling across storage, transportation, and wholesale. This stage requires highly portable, simple, affordable, and high-throughput detection technologies. Lateral flow strips, microfluidic chips, and low-cost sensors are therefore ideal. Pham et al. [43] replaced traditional antibodies with AuNP-aptamer conjugates, achieving visual detection of *Pseudomonas aeruginosa* within 15 min. Yang et al. [41] developed a reusable dual-mode aptasensor using bipolar electrodes. This device delivered a detection limit of 2.50 CFU/mL and a 3 min processing time. With a per-test cost below one US dollar and a matrix effect under 7%, the sensor is highly suitable for large-scale screening. Furthermore, Yin et al. [57] established a PMA-assisted digital CRISPR/Cas12a platform that operates without nucleic acid amplification. By achieving absolute quantification through single-molecule counting, the system bypasses the need for standard curves. It displayed a correlation coefficient of 0.9490 compared to traditional plate counting, providing a highly precise tool for the distribution stage. These innovations significantly lower the technical barrier for finished product testing and help safeguard the market supply.

### 8.4. Consumption Stage

The consumption stage focuses on rapid detection in households and restaurants, particularly for ready-to-eat foods. Assays for this setting demand exceptionally simple operations, intuitive results, and no professional training, with detection times strictly under 30 min. Zhang et al. [18] designed a one-pot platform using Cas13a and the fluorescent RNA aptamer Broccoli. By completely avoiding reverse transcription, amplification, and chemical labeling, the system allows consumers to visually observe fluorescence directly after adding a sample to the tube. Wu et al. [24] developed an epDNAzyme sequential reporting platform capable of detecting viable *Salmonella* at 190 CFU/mL in milk, beef, and blood within 25 min. The system successfully identified viable cell proportions as low as 0.1%. When coupled with a handheld fluorescence reader, this technology meets the strict speed and simplicity requirements of the consumption stage. Targeting *Staphylococcus aureus* in ready-to-eat foods, Geng et al. [50] combined dual aptamers with a CRISPR/Cas14 system. This assay achieved a sensitivity of 10 CFU/mL within 15 min at room temperature, entirely eliminating the need for prior nucleic acid extraction and amplification. Together, these technologies drive functional nucleic acid assays toward true sample-in-answer-out field applications, providing robust support for “last-mile” food safety.

## 9. Conclusions and Prospects

This review presents a systematic summary of molecular recognition strategies and detection techniques that use multiplexed FNAs to discriminate between viable and dead foodborne microorganisms. Based on their physicochemical properties and origins, the targeted biomarkers are classified into three major categories: nucleic acid-based, surface structure-related, and metabolic activity-linked indicators. The intrinsic mechanisms underlying viability discrimination for each category are detailed. Building on this classification, the core principles and technological evolution of primer nucleic acids, aptamers, DNAzymes, guide nucleic acids, and oligonucleotide probes are discussed. The progress of applying these technologies on-site across the four stages of the food supply chain (raw material harvest, processing, product distribution, and consumption) is also outlined, offering a technical reference for foodborne pathogen surveillance.

Overall, each category of FNA-based strategy has its distinct strengths and optimal application scenarios (Table 7). Primer-mediated amplification technologies offer the most mature performance and highest quantitative accuracy, making them the preferred choice for laboratory confirmation and regulatory testing. Aptamer-based platforms feature simple operation and no dependence on large instruments, and are most suitable for on-site rapid screening and large-scale batch inspection in resource-limited settings. DNAzyme systems achieve viability discrimination based on metabolic activity, providing a unique evaluation dimension for sterilization efficacy assessment in food processing. Guide nucleic acid-mediated technologies combine high programmability with signal amplification capacity, showing outstanding advantages in ultrasensitive and multiplex detection. Oligonucleotide probe methods enable high-throughput single-cell-level viability analysis and are ideal for viability monitoring of probiotics and fermented foods.

Future FNA-based viability detection technologies will increasingly integrate with emerging interdisciplinary fields to achieve higher sensitivity, multidimensional capability, and automation. For instance, combining artificial intelligence with high-throughput screening will accelerate the discovery and optimization of novel FNAs. Deep learning models can predict the three-dimensional conformations and binding affinities between aptamers and their targets. When paired with automated microfluidic SELEX platforms, these computational approaches reduce the screening time from months to days, improving the identification of low-abundance, unstable, or structurally complex biomarkers. Furthermore, machine learning-driven algorithms can efficiently process multidimensional data from complex sensor outputs, enabling accurate discrimination even at extremely low signal-to-noise ratios.

Transitioning these technologies to practical applications requires concurrent advancements in standardization and regulatory frameworks. Establishing a comprehensive reference material system that includes pure cultures, artificially contaminated foods, and authentic clinical samples is essential. This system would standardize the quantitative units for viable bacteria and calibrate them against FNA signal intensities. Organizations such as the International Organization for Standardization and Association of Official Agricultural Chemists should develop standardized protocols for FNA-based viability assays and promote their integration into food safety and clinical diagnostic regulations. Additionally, coupling these standardized methods with Internet of Things and blockchain technologies can facilitate the development of intelligent, self-reporting diagnostic terminals. These connected devices would enable end-to-end management, encompassing data collection, traceability, and early warning systems.

Beyond bacterial pathogens, FNA-based viability detection platforms also show promising potential for foodborne virus monitoring. By targeting viral genomic RNA, native envelope proteins, and host infectivity as viability biomarkers, FNA systems can be adapted to detect viable norovirus, hepatitis A virus, rotavirus and other common foodborne viruses, extending the technical support for full-spectrum food safety surveillance. The scope of viable bacteria detection is also expected to expand to include health indicators and ecologically relevant functional bacteria. Beyond traditional pathogens, FNAs can be engineered to monitor probiotic viability, track the activity dynamics of functional microbial communities in environmental settings, and analyze specific viable populations within the human microbiome. These applications extend the utility of FNA technologies into precision nutrition, environmental remediation, and personalized medicine. Ultimately, advancing FNA-based viability detection requires not only highly sensitive components and integrated devices but also an engineering approach to translate research findings into practical products. Systematic re-evaluation of viability criteria and interdisciplinary collaboration will be key to moving these diagnostic tools from the laboratory into routine use.

## Figures and Tables

**Table 1:** Comparison of traditional and FNA-based bacterial viability detection methods.

Detection Method	Detection Time	Analytical Sensitivity	Viable/Dead Discrimination	Cost per Test	Instrument Requirement	Typical Application Scenario	Main Limitations
Plate culture	3–5 days	Single CFU level	Yes	Low	Constant temperature incubator; basic laboratory conditions	Gold standard for laboratory confirmation and regulatory testing	Extremely long turnaround time; cumbersome operation; cannot meet rapid on-site detection demand
ELISA	2–4 h	10^3^–10^4^ CFU/mL	No	Medium	Microplate reader	High-throughput screening in food enterprises	Obvious batch-to-batch variation; unable to distinguish bacterial viability
Carbohydrate-binding module	1–3 h	10^2^–10^4^ CFU/mL	No	Medium	Microplate reader/fluorescence detector	Bacterial enrichment and polysaccharide-targeted detection in food matrices	Restricted to carbohydrate targets; lower affinity than antibodies; poor viability discrimination capacity
Conventional PCR	2–3 h	10–10^2^ CFU/mL	No	Medium	Thermal cycler/real-time fluorescence PCR instrument	Laboratory quantitative detection of bacterial nucleic acids	Overestimates viable counts due to dead bacterial DNA interference; highly dependent on precision instruments
FNA-based viability detection	15 min–3 h	Single CFU to 10^3^ CFU/mL	Yes	Low to medium	Flexible; supports instrument-free visual readout and laboratory precision detection	On-site rapid screening, laboratory high-sensitivity quantification, and full food supply chain monitoring	High-performance FNA screening is time-consuming; matrix tolerance in complex food samples needs further improvement

**Table 2:** Summary of primer nucleic acid-based viable/dead bacterial detection assays.

Functional Nucleic Acid	Target	Recognition Site	Signal Output	Sensitivity	Detection Range	Detection Time	Tool	Sample	Ref.
Primer	*Listeria monocytogenes*	*hly* gene	Colorimetric	3.5 × 10^3^ CFU/mL	10^0^~10^7^ CFU/mL	≤6 h	Test strip	Phosphate buffer, lettuce	[36]
	*Vibrio vulnificus*	*vvhA* gene	Fluorescent	29.33 CFU/mL	6.63 × 10^1^~1.33 × 10^6^ CFU/mL	3 h	Microfluidic chip	Plasma, pure culture	[28]
	*Lactic acid bacteria*	*pheS* gene	Fluorescent	10^6^ CFU/mL	10^6^~10^11^ CFU/mL	≤3 h	-	Fermented milk	[14]
	*Escherichia coli* O157:H7, *Staphylococcus aureus*, *Salmonella*	*rfbE*/*nuc*/*invA* gene	Fluorescent	10^4^ CFU/mL	10^1^~10^6^ CFU/mL	4~6 h	PCR tube	Milk, ground beef	[27]
	*Vibrio cholerae* O1/O139	*rfb*O1/*rfb*O139/*ctxA* gene	Fluorescent	102 CFU/mL	1.53 × 10^1^~1.53 × 10^6^ CFU/mL	3 h	Microfluidic chip	Seawater, pure culture	[15]
	*Salmonella*	*hilA* gene	Fluorescent	10 CFU	10^1^~10^6^ CFU	3 h	-	Milk, farm isolates	[20]
	Foodborne pathogenic bacteria	Genome	Fluorescent, colorimetric	-	-	-	-	Food samples	[26]
	*Listeria monocytogenes*	Genome	Fluorescent	-	-	-	-	Food samples	[37]
	Foodborne pathogenic bacteria	Nucleic acid	Fluorescent, colorimetric, electrochemical	-	-	-	-	Food, environmental samples	[38]
LAMP Primer	*Escherichia coli* O157:H7, *Salmonella*	DNA	Colorimetric	25 CFU	10^3^~10^8^ CFU/mL	2 h	Paper-based microfluidic chip	Foodborne pathogenic bacteria samples	[39]
RAA Primer	*Salmonella*	*invA* gene	Fluorescent	2.0 × 10^4^ CFU/mL	2 × 10^3^~2 × 10^7^ CFU/mL	1.5 h	-	Milk	[32]
RAA Primer	*Klebsiella pneumoniae*	*rcsA* gene	Fluorescent	2.6 × 10^3^ CFU/mL	2.6 × 10^2^~2.6 × 10^7^ CFU/mL	1.5 h	-	Infant formula	[33]
LAMP Primer	*Escherichia coli* O157:H7	*rfbE* gene	Colorimetric	81 CFU/g	10^0^~10^6^ CFU/g	2 h	Test strip	Lettuce	[13]
NASBA Primer	*Salmonella Enteritidis*	16S rRNA	Fluorescent	1.5 CFU	10^0^~10^8^ CFU	2.5 h	-	Rice, poultry, tap water, eggshell	[19]
LAMP Primer	*Salmonella*	*invA* gene	Colorimetric	1.22 CFU/mL	10^−1^~10^7^ CFU/mL	90 min	Filtration and concentration device	Onion, melon, salami	[40]
RAA Primer	*Salmonella* Typhimurium	DNA	Fluorescent	130 CFU/mL	1.3 × 10^2^~1.3 × 10^7^ CFU/mL	2 h	Microfluidic chip	Chicken	[35]
LAMP Primer	*Salmonella*	Nucleic acid	Colorimetric	-	-	-	Test strip	Pasture milk	[16]
Primer Nucleic Acid	Bacteria	Nucleic acid	Fluorescent, colorimetric	-	-	-	Portable detection device	Food, environmental samples	[34]
Primer Nucleic Acid	Foodborne pathogenic bacteria	Nucleic acid	Colorimetric	-	-	-	Test strip	Food	[31]

**Table 3:** Summary of aptamer-based viable/dead bacterial detection assays.

Functional Nucleic Acid	Target	Recognition Site	Signal Output	Sensitivity	Detection Range	Detection Time	Tool	Sample	Ref.
Aptamer	*Vibrio vulnificus*	Non-protein components of bacterial cell wall	Fluorescent	29.96 CFU/mL	10^2^–5 × 10^5^ CFU/mL	<1 h	-	Seawater, oyster, human serum	[44]
	*Staphylococcus aureus*	Viable bacterial surface	Fluorescent	400 CFU/mL	4 × 10^7^–400 CFU/mL	150 min	-	Tilapia blood, coconut water, milk	[21]
	*Salmonella* Typhimurium	Bacterial surface	Fluorescent	10^2^ CFU/mL	10^3^–10^7^ CFU/mL (BB-37); 10^2^–10^8^ CFU/mL (ROU-24)	30 min	-	Pork, egg white, milk, Chinese cabbage	[45]
	*Salmonella* Typhimurium	Viable bacterial surface	Colorimetric	1.2 CFU/mL	1–1.1 × 10^6^ CFU/mL	30 min	Portable chip	Skim milk	[23]
	*Staphylococcus aureus, Escherichia coli*	Viable bacterial surface	Fluorescent	10 CFU/mL	10^1^–10^5^ CFU/mL	15 min	-	Joint fluid (clinical sample)	[50]
	*Mycobacterium tuberculosis*	Secretory factor	Fluorescent	0.1 ag/mL	0.01 ag/mL–1 μg/mL	120 min	Smartphone	Human plasma	[52]
	*Pseudomonas aeruginosa*	LPS—universal polysaccharide antigen on bacterial surface	Colorimetric	2.34 × 10^2^ CFU/mL	10^1^–10^9^ CFU/mL	15 min	Test strip	Clinical specimen	[43]
	*Clostridium perfringens*	Bacterial surface	Electrochemical	2.50 CFU/mL	10^0^~10^8^ CFU/mL	3 min	Smartphone	Chicken breast, milk	[41]
	*Escherichia coli* O157:H7	Bacterial surface	Fluorescent	10 CFU/mL	10~10^5^ CFU/mL	3 h	-	Drinking water, river water, sewage treatment plant water	[22]

**Table 4:** Summary of DNAzyme-based viable/dead bacterial detection assays.

Functional Nucleic Acid	Target	Recognition Site	Signal Output	Sensitivity	Detection Range	Detection Time	Tool	Sample	Ref.
DNAzyme	*Escherichia coli* O157:H7, *Salmonella* Typhimurium	Bacterial surface	Fluorescent	50 CFU/mL	10^2^–10^8^ CFU/mL	30 min	Smartphone	Pork, milk	[53]
	*Salmonella Enteritidis*	Secretory factor	Fluorescent	190 CFU/mL	2–6 lg CFU/mL	25 min	-	Chicken, beef, milk, blood	[54]
	*Escherichia coli*	*phoA* gene	Colorimetric, electrochemical	<6.3 CFU/reaction	0.1 aM–10 fM	<2 h	Microfluidic chip	Leafy vegetables, milk	[55]
	*Escherichia coli*, *Staphylococcus aureus*, *Klebsiella pneumoniae*, *Salmonella* Typhimurium	Secretory protein	Fluorescent	1.3 × 10^3^ CFU/mL	1.5 × 10^3^–3.7 × 10^7^ CFU/mL	2.5 h	-	Fruit juice, milk, sputum	[24]
	*Salmonella Enteritidis*	Secretory factor	Photoelectrochemical	141 CFU/mL	2–7 lg CFU/mL	20 min	-	Beef, milk, blood	[25]

**Table 5:** Summary of guide nucleic acid-based viable/dead bacterial detection assays.

Functional Nucleic Acid	Target	Recognition Site	Signal Output	Sensitivity	Detection Range	Detection Time	Tool	Sample	Ref.
gRNA-CRISPR-Cas	*Bacillus cereus*, *Salmonella Enteritidis*, *Escherichia coli*	Bacterial RNA	Fluorescent	10 CFU	10~10^5^ CFU	20 min	-	Milk, rice	[18]
	*Viable Staphylococcus aureus*	Bacterial surface	Fluorescent	400 CFU/mL	400~4 × 10^7^ CFU/mL	150 min	-	Tilapia blood, coconut water, milk	[21]
	*Escherichia coli* O157:H7	Bacterial nucleic acid	Fluorescent	1.2 × 10^3^ CFU/mL	1.2 × 10^3^~1.2 × 10^7^ CFU/mL	120 min	Microfluidic chip	Water, milk, urine	[57]
	*Escherichia coli* O157:H7	Bacterial nucleic acid	Fluorescent	36 CFU/mL	36~10^7^ CFU/mL	30 min	Microfluidic chip	Water, milk	[58]
	*Puccinia striiformis f.* sp. *tritici*, *Barley stripe mosaic virus*, *Magnaporthe oryzae*	RNA	Bioluminescent	Sub-nanogram level	0.1%~100%	40 min	-	Wheat leaf	[62]
	*Lactobacillus*, *Viable Bacillus*	RNA	Fluorescent	1621 CFU/mL	1%~100%	30 min	-	Distiller’s yeast fermentation broth	[56]
	*Shigella flexneri*	Bacterial surface	Fluorescent	225 CFU/mL	2.25 × 10^3^~2.25 × 10^7^ CFU/mL	90 min	-	Milk, lake water, coconut water	[49]
gDNA-Ago	*Salmonella* Typhimurium	DNA	Fluorescent	23 CFU/mL	50~10^7^ CFU/mL	3.5 h	-	Pork, milk, fish, egg, orange juice	[59]
	*Salmonella*	DNA	Fluorescent	20 CFU/mL	10^2^~10^7^ CFU/mL	2 h	-	Chicken, pork, beef, egg	[61]
	*Salmonella* Typhimurium	DNA	Fluorescent	40.5 CFU/mL	50~10^7^ CFU/mL	3 h	-	Lettuce, pork, fish, chicken, milk, orange juice	[17]

**Table 6:** Summary of oligonucleotide probe-based viable/dead bacterial detection assays.

Functional Nucleic Acid	Target	Recognition Site	Signal Output	Sensitivity	Detection Range	Detection Time	Tool	Sample	Ref.
Oligonucleotide Probe	*Bacillus cereus*	16S rRNA	Fluorescent	10^2^ cells/g	10^2^~10^7^ cells/g	1.5 h	-	Milk powder, chicken breast	[66]
	*Lactobacillus*	16S rRNA	Fluorescent	2.7 × 10^4^ cells/g	10^4^~10^9^ cells/g	2.5 h	-	Infant formula	[65]
	*Listeria monocytogenes*	16S rRNA	Fluorescent	10^2^ cells/g	10^2^~10^8^ cells/g	2.5 h	-	Beef	[64]
	*Lactobacillus, Streptococcus thermophilus*	16S rRNA	Fluorescent	*Lactobacillus*: 2.42 × 10^4^ cells/mL; *Streptococcus thermophilus*: 1.67 × 10^4^ cells/mL	10^4^~10^8^ cells/mL	2 h	-	Yogurt, pork, milk, fish, egg, orange juice	[63]

**Table 7:** Comparison of five FNA-based viable bacteria detection strategies.

FNA Type	Core Recognition Mechanism	Viability Discrimination Principle	Typical Sensitivity	Detection Time	Optimal Application Scenario	Main Limitation
Primer nucleic acid	Complementary base pairing with target genomic/RNA sequences	Coupled with membrane dyes (PMA/PMAxx) or targeting short-lived mRNA	10–10^2^ CFU/mL	1.5–6 h	Laboratory high-sensitivity quantification, regulatory testing	Relies on pretreatment dyes; susceptible to amplification inhibitors in complex matrices
Aptamer	Spatial conformation binding to native surface antigens	Recognizes intact native conformations on viable cell surfaces; denatured antigens on dead cells cannot bind effectively	10–10^3^ CFU/mL	15 min–3 h	On-site rapid screening, high-throughput batch detection	High-affinity aptamer screening requires a certain cycle; matrix tolerance needs further optimization
DNAzyme	Catalytic cleavage triggered by metabolic enzymes or secretions	Targets active metabolic products exclusively secreted by viable bacteria	10^2^–10^3^ CFU/mL	20 min–2.5 h	Rapid evaluation of sterilization effect, on-site detection in processing links	Catalytic activity is slightly affected by environmental conditions; overall sensitivity is moderate
Guide nucleic acid	Programmable base pairing guided by gRNA/gDNA with effector proteins	Coupled with PMA for DNA targets, or inherent viability discrimination via RNA targeting	Single CFU to 10^2^ CFU/mL	20 min–3.5 h	Ultrasensitive multiplex detection, single-cell level quantification	Effector proteins have relatively high costs; reaction conditions are relatively strict
Oligonucleotide probe	Complementary hybridization with intracellular 16S rRNA	Relies on rapid degradation of rRNA in dead cells; can be coupled with PMA for dual verification	10^2^–10^4^ cells/g	1.5–2.5 h	High-throughput single-cell viability analysis, probiotic viability quantification	Requires professional flow cytometry operation; sensitivity is limited for ultra-low abundance samples

## Data Availability

No new data were created or analyzed in this study.
